# Antibiotic Resistance Profiling of *Staphylococcus aureus* Isolated from Clinical Specimens in a Tertiary Hospital from 2010 to 2012

**DOI:** 10.1155/2014/898457

**Published:** 2014-09-03

**Authors:** Alain C. Juayang, Gemma B. de los Reyes, April Joy G. de la Rama, Christine T. Gallega

**Affiliations:** ^1^Dr. Pablo O. Torre Memorial Hospital, 6100 Bacolod, Philippines; ^2^Colegio San Agustin, 6100 Bacolod, Philippines

## Abstract

MRSA infection can affect a wide array of individuals that may lead to treatment failure. Also, the infection has the potential to spread from one area to another particularly health care facilities or communities eventually causing minor outbreaks. With this premise, the study aimed to describe MRSA infections using the hospital-based data of a tertiary hospital in Bacolod City, Philippines, from 2010 to 2012. Specifically, this study aimed to evaluate the antimicrobial resistance of *S. aureus* isolated from clinical specimens and to put emphasis on the prevalence of MRSA and Inducible Clindamycin Resistance. A total of 94 cases from 2010 to 2012 were diagnosed to have *S. aureus* infection using conventional bacteriologic methods. From these cases, 38 (40.6%) were identified as MRSA and 37 (39.4%) were inducible clindamycin resistant. Wounds and abscesses were considered to be the most common specimens with MRSA infections having 71.05% while blood was the least with 5.3%. For drug susceptibility, out of the 94 *S. aureus* cases, including MRSA, 100% were susceptible to linezolid making it the drug of choice for this study. It was then followed by tetracycline having a mean susceptibility of 95%;, while penicillin G was ineffective with 94 cases having 0% susceptibility.

## 1. Introduction

*Staphylococcus aureus *is a common bacterium frequently isolated from the nares of humans [1]. It is also one of the most common pathogens that cause skin infections. When left untreated, disorders associated with this organism may progress in a wide range of conditions like tissue infections, pneumonia,wound, joint, and/or bone infections [2–4].

Most staphylococcal infections can be easily treated with antibiotics; however, in recent years* Staphylococcus* found its way to resist the commonly used and effective antibiotics; these antibiotics include macrolides, lincosamides, streptogramin, tetracycline, gentamicin, and beta-lactams particularly methicillin [2–6].

Methicillin-resistant* Staphylococcus aureus* (MRSA) was already present in the 1960s. After a decade, it was soon recognized as a serious nosocomial pathogen in the United States and has been an ever-growing problem worldwide [2–4, 6]. According to the paper of Delorme et al. [2], MRSA is already found in most individuals with apparently no age preferences. Moreover, Delorme et al. [2] noted that the infection has become common among admitted patients in hospitals and in nursing homes.

Also known as “a superbug,” MRSA has become a major problem in most medical institutions because it is creating life-threatening situations [7]. Furthermore, the emergence of mutated strains of MRSA is the vancomycin resistant* S. aureus* (VRSA) which has added peril to health care communities. VRSA is currently one of the greatest threats mankind faces because the antibiotic, vancomycin, is the last resort for treating staphylococcal infections [7].

The aims of this study are to evaluate the antimicrobial resistance of* S. aureus *isolated from clinical specimens of a tertiary hospital in Bacolod City and to highlight the pervasiveness of MRSA and Inducible Clindamycin Resistance (ICR) in this medical institution. For this reason, it is imperative for each area or region to be watchful for close monitoring and management.

## 2. Materials and Methods

Patterned from the study made by Delorme et al. [2] in 2009, this survey was performed in all cases of* Staphylococcus aureus* infections diagnosed by the pathology department of a tertiary hospital in Bacolod City, Philippines, from January 2010 to December 2012. A total of 94 cases were included in the study after a prospective review.

The diagnosis of* S. aureus* including methicillin-resistance and Inducible Clindamycin Resistance were determined based on the procedures by Forbes et al. [1] and recommendations from CLSI (2013) [8]. Among the antibiotics used were oxacillin (1 *µ*g), cefoxitin (30 *µ*g), erythromycin (15 *µ*g), clindamycin (2 *µ*g), azithromycin (15 *µ*g), penicillin G (10 units), ciprofloxacin (5 *µ*g), chloramphenicol (30 *µ*g), tetracycline (30 *µ*g), and linezolid (30 *µ*g). These antibiotics were included in the study because they are the recommended list of antibiotics to be routinely tested in the Performance Standards for Antibiotic Testing [8]. Methicillin-resistance was determined using the cefoxitin and oxacillin susceptibility, while Induced Clindamycin Resistance was performed using the* D*-Test. Isolates of* S. aureus* that are resistant to either cefoxitin or oxacillin were defined as MRSA, while those which are susceptible to both were classified as methicillin susceptible* Staphylococcus aureus* (MSSA). Also, the formation of* D*-shape zone of inhibition of clindamycin adjacent to erythromycin was noted to be an ICR positive [9].

The cases of infections were classified on the basis of methicillin-resistance, age, and types of specimen. Data were analyzed using WHONET 5.6 version downloaded from the World Health Organization (WHO) website. Age groups as pediatrics (<18 years old) and adults (≥18 years old) were automatically classified by the WHONET software.

## 3. Results

**Figure 1:**
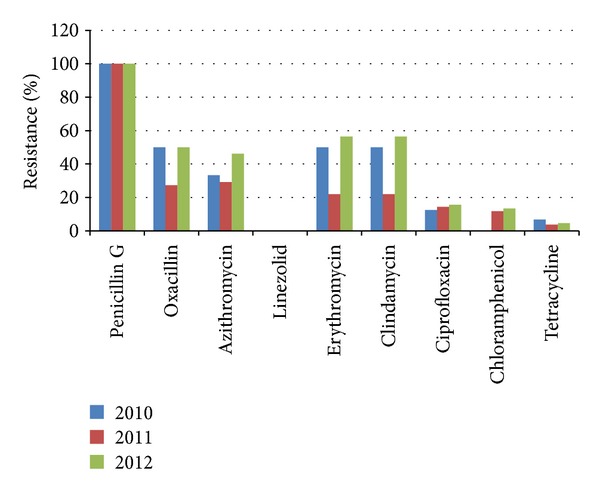
Percent of resistance of* S. aureus* including MRSA from 2010 to 2012.

For three years (2010, 2011, and 2012), there were 94 cases of* S. aureus* infections that were documented from the data base in the pathology department of a tertiary hospital in Bacolod City. Out of these 94 cases, 38 patients or 40.4% were documented as MRSA but the remaining 56 or 59.6% as MSSA.  Figure 1 illustrates MRSA cases based on their resistance to oxacillin and cefoxitin [8]; this also shows that ICR was found among the staphylococcal isolates to be as high with 53 (56.4%) isolates in 2012 utilizing the* D*-Test as a basis of interpretation [4, 8].

In vitro susceptibility test in Figure 1 shows that, out of the 9 antibiotics tested, linezolid worked best on all* S. aureus *isolates including MRSA while penicillin G was observed to be the least effective. In Table 1, a summary of MRSA and MSSA cases is presented; it was commonly found on wounds and abscesses as shown in Table 2. Consequently, Table 2 shows that, in this study, there were more adult patients infected with MRSA compared to pediatric patients. Additionally, there was neither significant difference on the age effect nor trend with regard to MRSA infection overtime in this study using Pearson Chi Square (*P* = 0.261) and Fisher's Exact Test (*P* = 0.331). From these 38 MRSA, a total of 36 isolates were found to be ICR positive, that is, 8 in 2010, 7 in 2011, and 21 in 2012. Among the 56 MSSA isolates, however, only 1 was found to be ICR positive and that was in 2012.

## 4. Discussions

*S. aureus* infections are generally common on skin lesions like wounds and other abscesses because this bacterium normally inhabits the skin [10]. Being an opportunistic organism,* S. aureus* proliferates easily and causes infections whenever it is transferred to other areas of the body or if a favorable environment for growth is present [1, 10, 11].

Further illustrated in this study is the susceptibility of* S. aureus* to linezolid. As shown in Figure 1, the 3-year data show linezolid to be the most effective drug, with a 0% resistance. Moreover, data of antibiotic susceptibility shows that the efficacy of penicillin G is exactly the opposite of linezolid that proves to be ineffective. Chloramphenicol, ciprofloxacin, and tetracycline show to be effective but with unwanted side effects [12]. Linezolid is the only drug in this study that is proven to be effective for both methicillin susceptible and -resistant* S. aureus*.

Vancomycin, the drug of choice for MRSA, was not tested because it may produce erratic results in disc diffusion susceptibility test [2]. However, even with the absence of vancomycin susceptibility test, the result of this study can be verified with the findings of several outcomes [7, 12–15] which elucidated that linezolid is a drug that is as effective as vancomycin. Both antibiotics do not just have similar failure and success rates but adverse effects as well [14, 15].

Table 1 also displays an increasing MRSA cases in three years with 25% to 50% against the total number of* S. aureus* infections. MRSA is considered to be a major nosocomial pathogen and has a high possibility of cross infection among patients and hospital staff according to Osawa et al. [16] and can cause significant morbidity and mortality [17]. In this study, MRSA is prominently found among adult patients and is commonly isolated on locations such as wounds and abscesses. These findings agreed with the data previously presented by Mehta et al. [18] and Tambekar et al. [17] which found a high prevalence of MRSA in wound sources. In Pakistan, Delorme et al. [2] also cited that the variations in MRSA resistance patterns often suggest that any strain of methicillin-sensitive* S. aureus* has the potential of becoming MRSA, noting that the evolution of and vertical transfer of resistance are a survival strategy of microorganisms [10].

Clindamycin is frequently used to treat skin and bone infections because of its patients' tolerability and excellent tissue penetration [19]. This antibiotic also accumulates in wound abscess [9] and is a good alternative treatment for MRSA and MSSA infections. Findings in this study, however, revealed an inducible* S. aureus* resistance to clindamycin as high as 56.4%. Even though clindamycin is a good antibiotic against staphylococcal infections, it cannot guarantee effective treatment because of high resistance rate [19].

## 5. Conclusions

In the light of the results of this study, the researchers concluded that less than 20% of* S. aureus *which were isolated mostly from the wounds and abscesses of admitted patients, of a tertiary hospital in Bacolod City, exhibited resistance to chloramphenicol, tetracycline, and ciprofloxacin but with excellent susceptibility to linezolid. The susceptibility test also confirms a high and increasing prevalence of MRSA and ICR among admitted patients. It is highly recommended that review of the existing preventive and control measures is done to arrest further escalation of cases that may pose greater human health threats in this medical institution.

## Figures and Tables

**Table 1:** Distribution of MRSA and MSSA isolated from clinical specimens.

	2010		2011		2012		Total	
	MRSA	MSSA	MRSA	MSSA	MRSA	MSSA	MRSA	MSSA
Wound and abscess	4	0	7	14	16	3	**27**	**17**
Blood	0	4	1	5	1	4	**2**	**13**
Respiratory Specimens	2	3	0	3	3	8	**5**	**14**
Urine	2	1	1	5	1	6	**4**	**12**
Total	**8**	**8**	**9**	**27**	**21**	**21**	**38**	**56**

**Table 2:** Age groups of patients infected with MRSA and MSSA.

	2010		2011		2012		Total	
	MRSA	MSSA	MRSA	MSSA	MRSA	MSSA	MRSA	MSSA
Pediatrics (<18 years old)	1	2	4	11	2	3	**7**	**16**
Adults (≥18 years old)	7	6	5	16	19	18	**31**	**40**
Total	**8**	**8**	**9**	**27**	**21**	**21**	**38**	**56**

## References

[1] Forbes B, Sahm D, Weissfeld A (2007). *Staphylococcus*, micrococcus and similar organisms. *Bailey and Scott’s Diagnostic Microbiology*.

[2] Delorme T, Rose S, Senita J, Callahan C, Nasr P (2009). Epidemiology and susceptibilities of methicillin-resistant Staphylococcus aureus in Northeastern Ohio. *The American Journal of Clinical Pathology*.

[3] Klein EY, Sun L, Smith DL, Laxminarayan R (2013). The changing epidemiology of methicillin-resistant *Staphylococcus aureus* in the United States: a national observational study. *The American Journal of Epidemiology*.

[4] Shen H, Akoda E, Zhang K (2013). Methicillin-resistant staphylococcus aureus carriage among students at a historically black university: a case study. *International Journal of Microbiology*.

[5] David MZ, Daum RS (2010). Community-associated methicillin-resistant *Staphylococcus aureus*: epidemiology and clinical consequences of an emerging epidemic. *Clinical Microbiology Reviews*.

[6] Gould IM (2005). The clinical significance of methicillin-resistant *Staphylococcus aureus*. *Journal of Hospital Infection*.

[7] Khan Z, Faisal S, Hasnain S (2010). The continuing threat of Methicillin Resistant *Staphylococcus aureus*—past, present, future. *Journal of Scientific Research*.

[8] Clinical and Laboratory Standards Institute (2013). Performance standards for antimicrobial disk susceptibility tests. *Approved Standard, Document M100-S23*.

[9] Yilmaz G, Aydin K, Iskender S, Caylan R, Koksal I (2007). Detection and prevalence of inducible clindamycin resistance in staphylococci. *Journal of Medical Microbiology*.

[10] Mahon C, Lehaman D, Manuselis G (2011). The Staphylococci. *Textbook of Diagnostic Microbiology*.

[11] Brooks G, Butel J, Carrol K, Mietzner T, Morse S (2010). The Staphylococci. *Jawetz, Melnick, & Aldenberg’s Medical Microbiology*.

[12] Gemmell CG, Edwards DI, Fraise AP, Gould FK, Ridgway GL, Warren RE (2006). Guidelines for the prophylaxis and treatment of methicillin-resistant *Staphylococcus aureus* (MRSA) infections in the UK. *Journal of Antimicrobial Chemotherapy*.

[13] Guzmán-Blanco M, Mejía C, Isturiz R (2009). Epidemiology of meticillin-resistant Staphylococcus aureus (MRSA) in Latin America. *International Journal of Antimicrobial Agents*.

[14] Pogue JM, Alaniz C (2012). Vancomycin versus linezolid in the treatment of methicillin-resistant staphylococcus aureus nosocomial pneumonia: implications of the ZEPHyR trial. *Annals of Pharmacotherapy*.

[15] Stevens DL, Herr D, Lampiris H, Hunt JL, Batts DH, Hafkin B (2002). Linezolid versus vancomycin for the treatment of methicillin-resistant *Staphylococcus aureus* infections. *Clinical Infectious Diseases*.

[16] Osawa K, Baba C, Ishimoto T (2003). Significance of methicillin-resistant *Staphylococcus aureus* (MRSA) survey in a university teaching hospital. *Journal of Infection and Chemotherapy*.

[17] Tambekar DH, Dhanorkar DV, Gulhane SR, Dudhane MN (2007). Prevalence and antimicrobial susceptibility pattern of methicillin resistant *Staphylococcus aureus* from healthcare and community associated sources. *African Journal of Infectious Disease*.

[18] Mehta AP, Rodrigues C, Sheth K, Jani S, Hakimiyan A, Fazalboy N (1998). Control of Methicillin Resistant *S. aureus* in tertiary care centre-a five year study. *Indian Journal of Medical Microbiology*.

[19] Zorgani A, Shawerf O, Tawil K, El-Turki E, Ghenghesh KS (2009). Inducible clindamycin resistance among staphylococci isolated from burn patients. *Libyan Journal of Medicine*.

